# Explaining Sentiment Classification with Synthetic Exemplars and Counter-Exemplars

**DOI:** 10.1007/978-3-030-61527-7_24

**Published:** 2020-09-19

**Authors:** Orestis Lampridis, Riccardo Guidotti, Salvatore Ruggieri

**Affiliations:** 8 University of Bari Aldo Moro, Bari, Italy; 9 Aristotle University of Thessaloniki, Thessaloniki, Greece; 10 Open University of Cyprus, Nicosia, Cyprus; 11 Dalhousie University, Halifax, NS Canada; 12 Aristotle University of Thessaloniki, Thessaloniki, Greece; 13 University of Pisa, Pisa, Italy; 14 ISTI-CNR, Pisa, Italy

**Keywords:** Explainable sentiment classification, Synthetic exemplars

## Abstract

We present xspells, a model-agnostic local approach for explaining the decisions of a black box model for sentiment classification of short texts. The explanations provided consist of a set of exemplar sentences and a set of counter-exemplar sentences. The former are examples classified by the black box with the same label as the text to explain. The latter are examples classified with a different label (a form of counter-factuals). Both are close in meaning to the text to explain, and both are meaningful sentences – albeit they are synthetically generated. xspells generates neighbors of the text to explain in a latent space using Variational Autoencoders for encoding text and decoding latent instances. A decision tree is learned from randomly generated neighbors, and used to drive the selection of the exemplars and counter-exemplars. We report experiments on two datasets showing that xspells outperforms the well-known lime method in terms of quality of explanations, fidelity, and usefulness, and that is comparable to it in terms of stability.

## Introduction

Opinions expressed by people in social media are increasingly being collected for several purposes [24]. People look at others’ opinions on a product before buying it, on a restaurant or hotel before making a reservation. Managers take decisions supported by consumers’ opinions on company brand, products, and services. Public decision makers care for what the citizens in their community want.

The massive amount of online texts (posts, tweets, reviews, etc.) makes it necessary to automate the analyses of such data. *Sentiment classification* is the task of learning a model that is able to predict the sentiment of a given text from labeled examples [31]. These machine learning models are exploited in various applications, e.g., personalization of advertisements, peer suggestion in social networks, recommendations of news, movies, etc. The analysis of short texts, which abound in micro-blogging sites such as Twitter and in online reviews, is especially challenging, due to their sparsity, non-uniformity, and noisiness. Deep Neural Networks (DNNs) [23, 40] and Random Forests (RFs) [6, 39], have been shown to be effective in terms of predictive accuracy and robustness to noise. However, the logic learned by a DNN or by a RF to classify a given text remains obscure to human inspection. These inscrutable “black box” models may hide biases learned from data, such as prejudice [2] or spurious correlations [33]. Consequently, they may reproduce and amplify such biases in their predictions [10].

Explainability of black box decisions is nowadays a mandatory requirement [9, 11]. Developers need to understand model’s decisions for debugging purposes. People subject to black box decisions may inquire to be provided with “meaningful information of the logic involved” (*right to explanation* [26] in the European Union GDPR). For example, if a comment in a social network has been removed because it has been classified as *hate speech*, the author has the right to know *why* the machine learning system has assigned such a label to her comment.

In this paper, we investigate the problem of explaining the decisions of a black box for sentiment classification on a given input (short) text. We design and experiment with a model-agnostic local approach named xspells (explaining sentiment prediction generating exemplars in the latent space). xspells’s explanations for the sentiment $y = b(x)$ assigned by a black box *b* to a text *x* consists of set of *exemplar* texts *E*, a set of *counter-exemplar* texts *C*, and the most frequent words in each of those sets $W = W_E \cup W_C$. Exemplars are sentences classified by the black box as *x* and close in meaning to *x*. They are intended to provide the user with hints about the kind of texts in the neighborhood of *x* that the black box classifies in the same way as *x*. Counter-exemplars are sentences that the black box classifies differently from *y*, but like exemplars, are also close in meaning to *x*. They are intended to provide the user with hints about the kind of texts in the neighborhood of *x* that the black box classifies differently from *x*. The usefulness of *counter-factual reasoning* has been widely recognized in the literature on explainable machine learning [4], particularly as a tool for causal understanding of the behavior of the black box. By contrasting exemplars and counter-exemplars, the user can gain an understanding of the factors affecting the classification of *x*. To help such an understanding, xspells provides also the most frequent words appearing in *E* and *C*.

The main novelty of our approach lies in the fact that the exemplars and counter-exemplars produced by xspells are *meaningful* texts, albeit synthetically generated. We map the input text *x* from a high-dimensional vector space into a low-dimensional latent space vector *z* by means of Variational Autoencoders [22], which couple encoding and decoding of texts. Then we study the behavior of the black box *b* in the neighborhood of *z*, or, more precisely, the behavior of *b* on texts decoded back from the latent space. Finally, we exploit a decision tree built from latent space neighborhood instances to drive the selection of exemplars and counter-exemplars. Experiments on two standard datasets and two black box classifiers show that xspells overtakes the baseline method lime [33] by providing understandable, faithful, useful, and stable explanations.

This paper is organized as follows. Section 2 discusses related work. Section 3 formalizes the problem and recalls key notions for the proposed method, which is described in Sect. 4. Section 5 presents an experimental validation. Finally, Sect. 6 summarizes our contribution, its limitations, and future work.

## Related Work

Research on interpretability and explainability in machine learning has bloomed over the last few years [17, 28]. Explanation methods can be categorized as: *(i)* *model-specific* or *model-agnostic*, depending on whether or not the approach requires access to the internals of the model; *(ii)* *local* or *global*, depending on whether the approach explains the prediction for a specific instance or the overall logic of the machine learning model.

xspells, falls into the category of *local*, *model-agnostic* methods which originated with [33] and extended along diverse directions by [12] and by [14, 16]. Well known model-agnostic local explanation methods able to also work on textual data include lime, anchor and shap. lime [33] randomly generates synthetic instances in the neighborhood of the instance to explain. An interpretable linear model is trained from such instances. Feature weights of the linear model are used for explaining the feature importance over the instance to explain. In the case of texts, a feature is associated to each word in a vocabulary. lime has two main weaknesses. First, the number of top features/words to be considered is assumed to be provided in input by the user. Second, the neighborhood texts are generated by randomly removing words, possibly generating meaningless texts [15]. anchor [34] is developed following principles similar to lime but it returns decision rules (called anchors) as explanations. It adopts a bandit algorithm that randomly constructs anchors with predefined minimum precision. Its weaknesses include the discretization of continuous features, the need for user-defined precision threshold parameters, and, as for lime, the usage of meaningless synthetic instances. shap [25] relates game theory with local explanations and overcomes some of the limitations of lime and anchor. Also shap audits the black box with possibly meaningless synthetic sentences. The method xspells proposed in this paper recovers from this drawback by generating the sentences for the neighborhood in a latent space by resorting to Variational Autoencoders.

lionets, deeplift and neurox are model-specific local explanation methods designed to explain deep neural networks able to work also on textual data. deeplift [36] decomposes the prediction of neural networks on a specific input by back-propagating the contributions of all neurons in the network to the input features. Then it compares the activation of each neuron to its “reference activation” and it assigns contribution scores according to the difference. neurox [7] facilitates the analysis of individual neurons in DNNs. In particular, it identifies specific dimensions in the vector representations learned by a neural network model that are responsible for specific properties. Afterwards, it allows the ranking of neurons and dimensions based on their overall saliency. Finally, lionets [29] looks at the penultimate layer of a DNN, which models texts in an alternative representation, randomly permutes the weights of nodes in that layer to generate new vectors, classifies them, observes the classification outcome and returns the explanation using a linear regressor like lime. Differently from these model-specific methods, xspells is not tied to a specific architecture and it can be used to explain any black box sentiment classifier.

## Setting the Stage

We address the *black box outcome explanation problem* [17] in the domain of sentiment classification, where machine learning classifiers are trained to predict the class value (sentiment) of a natural language text (simply, a text). We will mainly consider short texts such as posts on social networks, brief reviews, or single sentences, as these are typically the subject of sentiment classification. In this context, a black box model is a non-interpretable or inaccessible sentiment classifier *b* which assigns a sentiment label *y* to a given text *x*, i.e., $b(x) = y$. Example of black box models include Random Forests (RF) and Deep Neural Networks (DNN). We assume that the black box *b* can be queried at will. We use the notation *b*(*X*) as a shorthand for $\{b(x) \;|\; x \in X\}$. Formally, we have:

### Definition 1

Let *b* be a black box sentiment classifier, and *x* a text for which the decision $y=b(x)$ has to be explained. The *black box outcome explanation problem for sentiment classification* consists of providing an explanation $\xi \in \varXi$ belonging to a human-interpretable domain $\varXi$.

We introduce next the key tools that will be used in our approach.

### Factual and Counter-Factuals

A widely adopted human-interpretable domain $\varXi$ consists of *if-then* rules. They provide conditions (in the if-part) met by the instance *x* to be explained, that determined the answer of the black box (then-part). Rules can also be used to provide *counter-factuals*, namely alternative conditions, not met by *x*, that would determine a different answer by the black box [4]. In our approach, we will build on lore [14], a local explainer for *tabular data* that learns a decision tree from a given neighborhood *Z* of the instance to explain. Such a tree is a *surrogate* model of the black box, i.e., it is trained to reproduce the decisions of the black box. lore provides in output: *(i)* a *factual* rule *r*, corresponding to the path in the surrogate tree that explains why an instance *x* has been labeled as *y* by the black box *b*; and *(ii)* a set of *counter-factual* rules $\varPhi$, explaining minimal changes in the features of *x* that would change the class *y* assigned by *b*. In lore, the neighborhood *Z* is synthetically generated using a genetic algorithm that balances the number of instances similar to *x* and with its same label *y*, and the number of instances similar to *x* but with a different label $y' \ne y$ assigned by *b*.

### Variational Autoencoder

Local explanation methods audit the behavior of a black box in the neighborhood of the instance to explain. A non-trivial issue with textual data is how to generate *meaningful* synthetic sentences in the neighborhood (w.r.t. semantic similarity) of the instance. We tackle this problem by adopting Variational Autoencoders (VAEs) [22]. A VAE is trained with the aim of learning a representation that reduces the dimensionality from the large *m*-dimensional space of words to a small *k*-dimensional space of numbers (*latent space*), also capturing non-linear relationships. An *encoder* $\zeta$, and a decoder *decoder* $\eta$ are simultaneously learned with the objective of minimizing the *reconstruction loss*. Starting from the reduced encoding $z = \zeta (x)$, the VAE reconstructs a representation as close as possible to its original input $\tilde{x} = \eta (z) \simeq x$. After training, the *decoder* can be used with generative purposes to reconstruct instances never observed by generating vectors in the latent space of dimensionality *k*. The difference with standard autoencoders [19] is that VAEs are trained by considering an additional limitation on the loss function such that the latent space is scattered and does not contain “dead zones”. Indeed, the name *variational* comes from the fact that VAEs work by approaching the posterior distribution with a variational distribution. The encoder $\zeta$ emits the parameters for this variational distribution, in terms of a multi-factorial Gaussian distribution, and the latent representation is taken by sampling this distribution. The decoder $\eta$ takes as input the latent representation and focuses on reconstructing the original input from it. The avoidance of dead zones ensures that the instances reconstructed from vectors in the latent space, e.g., posts or tweets, are semantically meaningful [3].
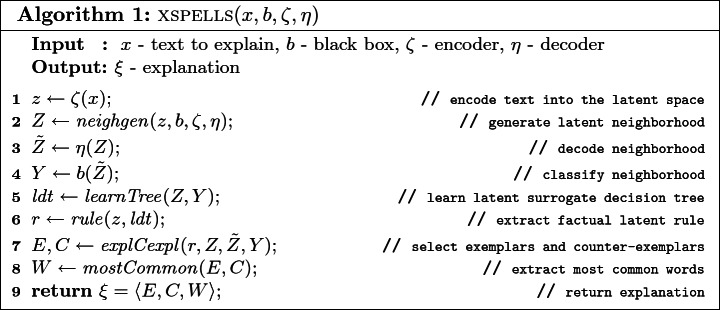


## Explaining Sentiment Classifiers

We propose a local model agnostic explainer for sentiment classification of short texts, called xspells (*e* x*plaining* s*entiment* p*rediction generating* e*xemp* l*ars in the* l*atent* s*pace*). Given a black box *b*, a short text *x*, e.g., a post on a social network, and the sentiment label $y = b(x)$ assigned by the black box, e.g., *hate* or *neutral*, the explanation provided by xspells is composed of: *(i)* a set of *exemplar* texts; *(ii)* a set of *counter-exemplar* texts; and, *(iii)* the set of *most common words* in exemplars and counter-exemplars. Exemplar and counter-exemplar texts respectively illustrate instances classified with the same and with a different label than *x*. Such texts are close in meaning to *x*, and they offer an understanding of what makes the black box determine the sentiment of texts in the neighborhood of *x*. Exemplars help in understanding reasons for the sentiment assigned to *x*. Counter-exemplars help in understanding reasons that would reverse the sentiment assigned. The most common words in the exemplars and counter-exemplars may allow for highlighting terms (not necessarily appearing in *x*) that discriminate between the assigned sentiment and a different sentiment. These components form the human-interpretable explanation $\xi \in \varXi$ for the classification $y = b(x)$ returned by xspells, whose aim is to satisfy the requirements of counter-factuability, usability, and meaningfulness [4, 28, 32].

**Fig. 1.**
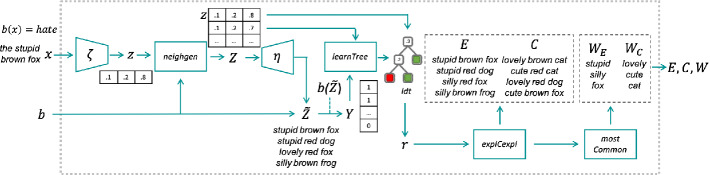
xspells process on a sample input. xspells takes as input the short text *x* and the sentiment assigned *b*(*x*). The output is a set of exemplars and counter-exemplars, and the most common discriminative words.

Besides the black box *b* and the text *x* to explain, xspells is parametric in: an encoder $\zeta$ and a decoder $\eta$ for representing texts in a compact way in the latent space. Algorithm 1 details xspells, and Fig. 1 shows the steps of the explanation process on a sample input. First, *x* is transformed into a low-dimensionality vector $z = \zeta (x)$ in the latent space. xspells then generates a neighborhood *Z* of *z*, which is decoded back to a set of texts $\tilde{Z}$. The dataset *Z* and the decisions of the black box on the decoded text $Y = b(\tilde{Z})$ are used to train a surrogate decision tree (in the latent space).

Then, the $$ explCexpl ()$$ module selects exemplars *E* and counter-exemplars *C* from *Z* by exploiting the knowledge extracted (i.e., the decision tree branches), and decodes them into texts. Finally, the most common words $$W = W_E \cup W_C$$ are extracted from *E* and *C* and the overall explanation $$\xi $$ is returned. Details of each step are presented in the rest of this section.

### Latent Encoding and Neighborhood Generation

The input text *x* is first passed to a trained VAE $\zeta$ (line 1 of Algorithm 1), thus obtaining the latent space representation $z = \zeta (x)$. The number of latent dimensions *k* is kept low to avoid dimensionality problems. We capture the sequential information in texts by adopting VAEs based on long short-term memory layers (LSTM) [20] for both the encoder $\zeta$ and decoder $\eta$ (lines 1 and 3). In particular, the decoder $\eta$ is trained to predict the next characters of the text, given the previous characters of the text. In more detail, it is trained to convert a given text into the same text, but being offset by a time-step in the future.

xspells generates a set *Z* of *n* instances in the latent feature space for a given *z*. The neighborhood generation function $neighgen$ (line 2) can be implemented by adopting several different strategies, ranging from a purely random approach like in lime [33], to using a given distribution and a genetic algorithm maximizing a fitness function like in lore [14]. xspells adopts a random generation of latent synthetic instances by relying on the fact that the encoder maps uniformly the data distribution over the latent space. xspells guarantees a minimum number *n* of distinct instances by removing duplicates. Next, xspells uses the synthetically generated instances $\tilde{Z}$ for querying the black box *b* (line 4). This is made possible by turning back the latent representation to text through the decoder $\eta$ [3] (line 3). We tackle the requirement of generating *local* instances by randomly generating $N \gg n$ latent instances, and then retaining in *Z* only the *n* closest instances to *z*, i.e., $|Z| = n$. The distance used in the latent space is the Euclidean distance. The neighborhood generation $neighgen$ actually returns a set $Z = Z_{=} \cup Z_{\ne }$ with $z' \in Z_=$ such that $b(\eta (z')) = b(\eta (z))$, and instances $z' \in Z_{\ne }$ such that $b(\eta (z')) \ne b(\eta (z))$. We further consider the problem of imbalanced distributions in *Z*, which may lead to weak decision trees. Class balancing between the two partitions is achieved by adopting the SMOTE [5] procedure if the proportion of the minority class is less than a predefined threshold $\tau$.

### Local Latent Rules and Explanation Extraction

Given *Z* and $Y = b(\tilde{Z})$, xspells builds a latent decision tree $ldt$ (line 5) acting as a local surrogate of the black box, i.e., being able to locally mime the behavior of *b*. xspells adopts decision tree because decision rules can be derived from a root-to-leaf path [14]. Indeed, the premise *p* of the rule $r = p \,{\rightarrow }\, y$ is the conjunction of the split conditions from the root to the leaf of the tree that is followed by features in *z*. This approach is a variant of lore (see Sect. 3.1) but in a latent feature space. The consequence *y* of the rule is the class assigned at that leaf^1^.

Given a text *x*, the explanations returned by xspells are of the form $\xi =\langle E, C, W \rangle$, where: $E = \{e^x_1, \dots , e^x_u\}$ is the set of *exemplars* ($b(e^x_i) = b(x) \; \forall i \in [1, u]$); $C = \{c^x_1, \ldots , c^x_v\}$ is the set of *counter-exemplars* ($b(c^x_i) \ne b(x) \; \forall i \in [1, v]$); and $W = W_E \cup W_C$ is the set of the *h* most frequent words in exemplars *E* and of the *h* most frequent words in counter-exemplars *C*. Here, *u*, *v*, and *h* are parameters that can be set in xspells. Exemplars are chosen starting from the latent instances in *Z* which satisfy both the premise *p* and the consequence *y* of the rule $r = p \rightarrow y$ above, namely the instances $z' \in Z$ that follow the same path as *z* in the decision tree, and such that the $b(\eta (z')) = y$. The *u* instances $z'$ closest to *z* are selected, using Euclidean distance. They are decoded back to the text space $\eta (z')$ and included in *E*. Counter-exemplars are chosen starting from the latent instances $z' \in Z$ which do not satisfy the premise *p* and such that $b(\eta (z')) \ne b(x)$. The *v* instances closest to *z* are chosen. They are decoded back to the text space $\eta (z')$ and included in *C*.

## Experiments

In this section, we illustrate qualitative/quantitative experimental analyses of faithfulness, usefulness, and stability properties of xspells explanations^2^. The xspells system has been developed in Python, and it relies on the CART decision tree algorithm as implemented by the scikit-learn library, and on VAEe implemented with the keras library^3^.

### Experimental Settings

We experimented with the proposed approach on two datasets of tweets. The *hate speech dataset* (hate) [8] contains tweets labeled as hate, offensive or neutral. Here, we focus on the 1,430 tweets that belong to the *hate* class, and on the 4,163 tweets of the *neutral* class. The *polarity dataset* (polarity) [30] contains tweets about movie reviews. Half of these tweets are classified as *positive* reviews, and the other half as *negative* ones. These two datasets are remarkable examples where a black box approach is likely to be used to remove posts or to ban users, possibly in automated way. Such extreme actions risk to hurt the free speech rights of people. Explanations of the black box decision are then of primary relevance both to account for the action and to test/debug the black box.

For both datasets, we use 75% of the available data for training a black box machine learning classifier. The remaining 25% of data is used for testing the black box decisions. More specifically, 75% of that testing data is used for training the autoencoder, and 25% for explaining black box decisions (*explanation set*). Datasets details are reported in Table 1 (left).

**Table 1.** Datasets description, black box models accuracy, and VAE RMSE.

Dataset	No. tweets	Avg. no words	No. classes	Bb train size	VAE train size	Expl. size	Accuracy		VAE
							RF	DNN	MRE
hate	5,593	20.82	2	4,195	1,048	350	.9257	.8485	0.26
polarity	10,660	24.87	2	7,995	1,998	666	.6702	.6302	0.59

We trained and explained the following black box classifiers: Random Forest [38] (RF) as implemented by the scikit-learn library, and Deep Neural Networks (DNN) implemented with the keras library. For the RF, we transformed texts into their TF-IDF weight vectors [38], after removing stop-words, including Twitter stop-words such as “rt”, hashtags, URLs and usernames. A randomized cross-validation search was then performed for parameter tuning. Parameters for RF models were set as follows: 100 decision trees, *Gini* split criterion, $\sqrt{m}$ random features where *m* is the total number of features; no limit on tree depth. The DNNs adopted have the following architecture. The first layer is a dense embedding layer. It takes as input a sparse vector representation of each text (subject to same pre-processing steps as for the RF, without the TF-IDF representation) obtained by using a Keras tokenizer^4^ to turn the text into an array of integers and a padder so that each vector has the same length. This way, we allow the network to learn its own dense embeddings of size 64. The first embedding layer is followed by a dropout layer at 0.25. Afterwards, the DNN is composed by three dense layers with sizes 64, 512 and 128. The central layer is an LSTM [20] that captures the sequential nature of texts and has size 100. After that, there are three dense layers with sizes 512, 64 and 32. The dense layers adopt the *ReLu* activation function. Finally, the *sigmoid* activation function is used for the final classification. We adopted *binary cross-entropy* as loss function and the *Adam* optimizer. We trained the DNN for 100 epochs. Classification performances are reported in Table 1 (center-right).

We designed the VAEs used in experiments with both the encoder $\zeta$ and the decoder $\eta$ consisting of a single LSTM layer. We fed the text into the VAE using a one-hot vectorization that takes an input tensors with dimensions $33 \cdot 5368 \,{=}\, 177,144$ for the hate dataset, and $48 \cdot 5308 \,{=}\, 254,784$ for the polarity dataset, after stop-words removal. The numbers above represent the maximum text length and the number of distinct words considered. In order to provide to the VAE knowledge also about unseen words with respect to those in its training set, we extended the vocabulary with the 1000 most common English words^5^ We considered $k\,{=}\,500$ latent features for both datasets^6^. Table 1 (right) reports the *Mean Reconstruction Error* (MRE) calculated as the average cosine similarity distance between the original and reconstructed texts when converted to TF-IDF vectors. We set the following xspells hyper-parameters. The neighborhood generation $neighgen$ is run with $N \,{=}\, 600$, $n \,{=}\, 200$, $\tau \,{=}\,40\%$. For the latent decision tree we used the default parameter of the CART implementation. Finally, with regards to the explanation hyper-parameters, we set $u\,{=}\,v\,{=}\,5$ (counter-)exemplars, and $h\,{=}\,5$ most frequent words for exemplars and for counter-exemplars.

In the experiments we compare xspells against lime [33]. We cannot compare against shap [25] and anchor [34] because it is not immediate how to practically employ them to explain sentiment classifiers. Other approaches such as IntGrad [37] or LRP [1] could theoretically be used to explain sentiment classifiers. However, first, they are not agnostic but tied to DNNs, and second, they are typically used for explaining image classifiers.

**Table 2.** Explanations returned by xspells for texts classified as *hate* in the hate dataset, and as *negative* in the polarity dataset. Three exemplars (E) and two counter-exemplars (C) for each tweet. Relative word frequencies in parenthesis.

	Tweet	(Counter-)exemplars	E/C	$W_{=}$	$W_{\ne }$
Hate	I dont have any problems with zak, but you seem like a faggot	I hate dumb bitches	E	Hate (.22)	Work (.06)
		I hate fat bitches wear show	E	Bitches (.17)	Love (.06)
		I hate fat bitches	E	Fat (.11)	Wearing (.06)
		This is why i work	C	Dumb (.06)	Fuzzy (.06)
		I really want a girl	C	Wear (.06)	Blankets (.06)
hate	California’s biggest retards. Don’t forget about HOLY who just released an amazing EP	This girl is retarded	E	Retarded (.08)	Im (.14)
		The fucking royals bitch work	E	Hated (.08)	Love (.07)
		Im such a retard sometimes	E	Bitch (.08)	Birds (.07)
		This is why i love birds	C	Fucking (.08)	Brownies (.07)
		Wait did take my brownies	C	Retard (.08)	Sorry (.07)
polarity	Eccentric enough to stave off doldrums, caruso’s self-conscious debut is also eminently forgettable	It has ever under trash without to a familiar	E	Trash (.05)	Fun (.10)
		This extremely unfunny movie in at 80 min	E	Imperfect (.05)	Remarkable (.07)
		This movie makes for one thing imperfect	E	Unfunny (.05)	Appears (.07)
		A story of musical and character and love	C	Without (.05)	Want (.04)
		It is a movie fun for fans who cant stop	C	Ever (.05)	Love (.04)
polarity	While some of the camera work is interesting, the film’s mid-to-low budget is betrayed by the surprisingly shoddy makeup work	In the end i kept this one at two stars	E	Bad (.07)	New (.12)
		Odd poetic road movie spiked by jolts of pop	E	Attempt (.07)	Really (.06)
		In attempt to the bad sense with this summer	E	End (.07)	Safe (.03)
		Does what a fine documentary does best	C	Sense (.04)	Fine (.03)
		A film that plays things so nice n safe	C	Odd (.04)	Safe (.03)

### Qualitative Evaluation

In this section, we qualitatively compare xspells explanations with those returned by lime. Tables 2 and 3 show sample explanations for both experimental datasets, and considering the RF black box sentiment classifier.

The first and second tweet in Table 2 belong to the hate dataset and are classified as *hate*. Looking at the exemplars returned by xspells, the *hate* sentiment emerges from the presence of the word “hate”, from sexually degrading references, and from derogatory adjectives. On the other hand counter-exemplars refer to women and to work with a positive perspective. The second tweet for the hate dataset follows a similar pattern. The focus this time is on the word “retard”, used here with negative connotations. Differently from xspells, the explanations returned by lime in Table 3 for the same tweets show that the hate sentiment is mainly due to the words “faggot” and “retards” but there are not any further details, hence providing to the user a limited understanding.

**Table 3.** Explanations returned by lime for tweets classified as *hate* in the hate dataset, and as *negative* in the polarity dataset. lime word importance in parenthesis.

	Tweet	Top features		Tweet	Top features
hate	I dont have any problems with zak, but you seem like a faggot	Faggot (−0.62)	polarity	Eccentric enough to stave off doldrums, caruso’s self-conscious debut is also eminently forgettable	off (−0.30)
		You (−0.03)			Debut (0.03)
		Like (0.01)			Enough (0.03)
		Any (−0.01)			Also (0.03)
		Problems (0.01)			Self (−0.01)
hate	California’s biggest retards. Don’t forget about HOLY who just released an amazing EP	Retards (−0.24)	polarity	While some of the camera work is interesting, the film’s mid-to-low budget is betrayed by the surprisingly shoddy makeup work	Work (0.11)
		Dont (−0.03)			While (0.04)
		California (−0.01)			low (−0.04)
		Who (−0.01)			Some (−0.04)
		Holy (0.01)			Interesting (−0.03)

The usefulness of the exemplars and counter-exemplars of xspells are even more clear for the polarity dataset, where the RF correctly assigns the sentiment *negative* to the sample tweets in Table 2. For the first tweet, xspells recognizes the negative sentiment captured by the RF and provide exemplars containing negative words such as “trash”, “imperfect”, and “extremely unfunny” as negative synonyms of “eccentric”, “forgettable”, and “doldrums”. The counter-exemplars show the positive connotation and context that words must have to turn the sentiment into *positive*. On the contrary, lime (Table 3) is not able to capture such complex words and it focuses on terms like “off”, “debut”, or “enough”. For the second tweet, xspells is able to generates exemplar similar in meaning to the tweet investigated: the tweet starts positive (or appear positive), but reveals/hides a negative sentiment in the end. In this case the most frequent words alone are not very useful. Indeed, (the surrogate linear classifier of) lime mis-classifies the second tweet as positive giving importance to the word “work” that, however, is not the focus of the negative sentiment.

Overall, since lime extracts words from the text under analysis, it can only provide explanations using such words. On the contrary, the (counter-)exemplars of xspells consist of texts which are close in meaning, but including different wordings that help the user better grasp the reasons behind black box decision.

**Table 4.** Mean and standard deviation of fidelity. The higher the better.

	RF		DNN	
	lime	xspells	lime	xspells
hate	0.62 ± 0.30	$\underline{0.98 \pm 0.01}$	0.92 ± 0.15	0.98 ± 0.01
polarity	0.89 ± 0.14	$\underline{0.98 \pm 0.01}$	0.91 ± 0.20	$\underline{0.97 \pm 0.01}$

### Fidelity Evaluation

We evaluate the *faithfulness* [11, 17] of the surrogate latent decision tree adopted by xspells by measuring how well it reproduces the behavior of the black box *b* in the neighborhood of the text *x* to explain – a metric known as *fidelity*. Let *Z* be the neighborhood of *x* in the latent space generated at line 2 of Algorithm 1 and $ldt$ be the surrogate decision tree computed at line 5. The fidelity metric is $|\{ y \in Z \ |\ ldt (y) = b(\eta (y)) \}|/|Z|$, namely the accuracy of $ldt$ assuming as ground truth the black box. The fidelity values over all instances in the explanation set are aggregated by taking their average and standard deviation.

We compare xspells against lime, which adopts as surrogate model a linear regression over the feature space of words and generates the neighborhood using a purely random strategy. Table 4 reports the average fidelity and its standard deviation. On the hate dataset, xspells reaches almost perfect fidelity for both black boxes. lime performances are markedly lower for the RF black box. On the polarity dataset, the difference is less marked, but still in favor of xspells. A Welch’s t-test shows that the difference of fidelity between xspells and lime is statistically significant (p-value $< 0.01$) in all cases from Table 4.

### Usefulness Evaluation

How can we evaluate the usefulness of xspells explanations? The gold standard would require to run lab experiments involving human evaluators. Inspired by [21], we provide here an indirect evaluation by means of a k-Nearest Neighbor (k-NN) classifier [38]. For a text *x* in the explanation set, first we randomly select *n* exemplars and *n* counter-exemplars from the output of xspells. Then, a 1-NN classifier^7^ is trained over such (counter-)exemplars. Finally, we test 1-NN over the text *x* and compare the prediction of 1-NN with the sentiment *b*(*x*) predicted by the black box. In other words, the 1-NN approximates a human in assessing the (counter-)exemplars usefulness. The accuracy computed over all *x*’s in the explanation set is a proxy measure of how good/useful are (counter-)exemplars at delimiting the decision boundary of the black box. We compare such an approach with a *baseline* (or null) model consisting of a 1-NN trained on *n* texts per sentiment, selected randomly from the training set and not including *x*.

**Fig. 2.**
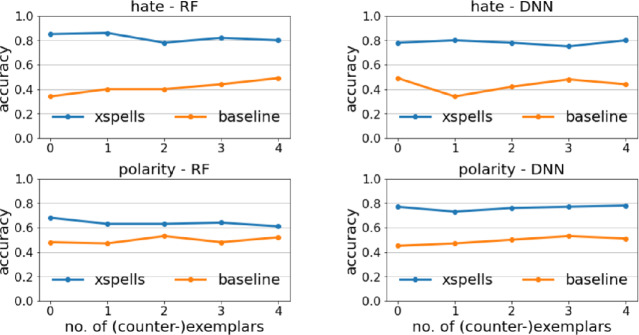
Usefuless as 1-NN accuracy varying the number of (counter-)exemplars.

The accuracy of the two approaches are reported in Fig. 2 by varying the number *n* of exemplars and counter-exemplars. xspells neatly overcomes the *baseline*. The difference is particularly marked for when *n* is small. Even though the difference tend to decrease for large *n*’s, large-sized explanations are less useful in practice due to cognitive limitations of human evaluators. Moreover, xspells performances are quite stable w.r.t. *n*, i.e., even one or two exemplars and counter-exemplars are sufficient to let the 1-NN classifier distinguish the sentiment assigned to *x* in an accurate way.

**Table 5.** Mean and stdev of the coherence index $\mathcal {C}_x$. The closer to 1 the better.

	RF		DNN	
	lime	xspells	lime	xspells
hate	1.10 ± 0.17	1.05 ± 0.25	1.06 ± 0.08	1.12 ± 0.39
polarity	1.05 ± 0.15	1.15 ± 0.20	1.13 ± 0.18	1.09 ± 0.14

### Stability Evaluation

Stability of explanations is a key requirement, which heavily impacts users’ trust on explainability methods [35]. Several metrics of stability can be devised [18, 27]. A possible choice is to use sensitivity analysis with regard to how much an explanation varies on the basis of the randomness in the explanation process. Local methods relying on random generation of neighborhoods are particularly sensitive to this problem. In addition, our method suffers of the variability introduced by the encoding-decoding of texts in the latent space. Therefore, we measure here stability as a relative notion, that we call *coherence*. For a given text *x* in the explanation set, we consider its closest text $x^c$ and its *k*-th closest text $x^f$, again in the explanation set. A form of Lipschitz condition [27] would require that the distance between the explanations *e*(*x*) and $e(x^f)$, normalized by the distance between *x* and $x^f$, should not be much different than the distance between the explanations *e*(*x*) and $e(x^c)$, again normalized by the distance between *x* and $x^c$. Stated in words, normalized distances between explanations should be as similar as possible. Formally, we introduce the following *coherence index*:

$$\begin{aligned} \mathcal {C}_x = \frac{ dist _e(e({x^{f})}, e({x}))/ dist (x^{f}, x)}{ dist _e(e({x^{c}}), e({x}))/ dist (x^{c}, x)} \end{aligned}$$

where we adopt as distance function $$ dist $$ the cosine distance between the TF-IDF representation of the texts, and as distance function $$ dist _e$$ the Jaccard distance between the 10 most frequent words in each explanation (namely, the *W* set). In experiments, we set $$x^f$$ to be the $$k=10$$-closest text w.r.t. *x*. For comparison, the coherence index is computed also for lime, with Jaccard similarity calculated between the sets of 10 words (a.k.a. features) that lime deems more relevant.

Table 5 reports the average coherence over the explanation set. xspells and lime have comparable levels of coherence, and an even number of cases where one overcomes the other. A Welch’s t-test shows that the difference of the coherence indexes between xspells and lime is statistically significant (p-value $< 0.01$) in only one case, namely for the polarity dataset and RF black box model.

## Conclusion

We have presented xspells, a local model-agnostic explanation approach for black box sentiment classifiers. The key feature of xspells is the adoption of variational autoencoders for generating meaningful synthetic texts from a latent space. Such a space reveals essential also for inducing a decision tree which helps in characterizing exemplar and counter-factual exemplar texts. The approach advances over baseline explainers, such as lime, which only highlight the contribution of words already in the text to explain. Experiments showed that xspells also exhibits better fidelity and usefulness, and comparable stability.

The proposed approach has some clear limitations. *First*, performance is strictly dependent on the VAE adopted: a better autoencoder would lead to more realistic exemplars and counter-exemplars. The structure of the autoencoder needs then to be further explored and evaluated beyond the specific one adopted in this paper. This may also require trading-off quality with computational costs, which may slow down the response time of xspells. *Second*, we will consider extending the explanations returned by xspells with logic rules, which convey information at a more abstract level than exemplars. Such rules can be extracted from the decision tree on the latent space, but have to decoded back to rules on texts – a challenging task. *Third*, xspells could be extended to account for long texts, e.g., by adopting word2vec embeddings [13] for modeling the input/output of the VAE. *Fourth*, we could rely on linguistic resources, such a thesaurus or domain ontologies, to empower both synthetic text generation and to enrich the expressiveness of the (counter-)exemplars. *Fifth*, a human evaluation of xspells would be definitively required, e.g., through crowdsourcing experiments.

## Footnotes

1 In theory, it might happen that $y \ne b(x)$, namely the path followed by *z* predicts a sentiment different from *b*(*x*). In our experiments, this never occurred. In such cases, xspells restarts by generating a new neighborhood and then a new decision tree.

2 The source code is available at: *https://github.com/orestislampridis/X-SPELLS*.

3 https://scikit-learn.org/stable/modules/tree.html, https://keras.io.

4 https://keras.io/preprocessing/text.

5 https://1000mostcommonwords.com.

6 Experiments (not reported due to lack of space) show that $k=500$ is a good compromise between MRE and the reduced dimensionality of the latent space when varying $k \in \{100, 250, 500, 1000, 2500\}$.

7 Distance function adopted: cosine distance between the TF-IDF representations.
